# Enantioselective Phytotoxicity of the Herbicide Imazethapyr on the Response of the Antioxidant System and Starch Metabolism in *Arabidopsis thaliana*

**DOI:** 10.1371/journal.pone.0019451

**Published:** 2011-05-06

**Authors:** HaiFeng Qian, Tao Lu, XiaoFeng Peng, Xiao Han, ZhengWei Fu, WeiPing Liu

**Affiliations:** College of Biological and Environmental Engineering, Zhejiang University of Technology, Hangzhou, People's Republic of China; Texas A&M University, United States of America

## Abstract

**Background:**

The enantiomers of a chiral compound possess different biological activities, and one of the enantiomers usually shows a higher level of toxicity. Therefore, the exploration of the causative mechanism of enantioselective toxicity is regarded as one of primary goals of biological chemistry. Imazethapyr (IM) is an acetolactate synthase (ALS)-inhibiting chiral herbicide that has been widely used in recent years with racemate. We investigated the enantioselectivity between *R*- and *S*-IM to form reactive oxygen species (ROS) and to regulate antioxidant gene transcription and enzyme activity.

**Results:**

Dramatic differences between the enantiomers were observed: the enantiomer of *R*-IM powerfully induced ROS formation, yet drastically reduced antioxidant gene transcription and enzyme activity, which led to an oxidative stress. The mechanism by which IM affects carbohydrate metabolism in chloroplasts has long remained a mystery. Here we report evidence that enantioselectivity also exists in starch metabolism. The enantiomer of *R*-IM resulted in the accumulation of glucose, maltose and sucrose in the cytoplasm or the chloroplast and disturbed carbohydrates utilization.

**Conclusion:**

The study suggests that *R*-IM more strongly retarded plant growth than *S*-IM not only by acting on ALS, but also by causing an imbalance in the antioxidant system and the disturbance of carbohydrate metabolism with enantioselective manner.

## Introduction

Pesticides are commonly applied to crops in order to control insects, disease and weeds. It has been estimated that more than 40% of currently used pesticides in China are chiral compounds [1], and the proportion is expected to increase as compounds with more complex structures synthesized. However, because of their similar physical-chemical properties, few enantiomers of chiral pesticide are used separately. Over the past two decades, many reports have been demonstrated that enantimers differ in biological properties including biodegradation [2], [3], acute or chronic toxicity [4], [5], developmental toxicity [6] and endocrine-disrupting activities [7]–[9].

Imidazolinone (IMI) herbicides are commonly applied as either pre- or post-emergence herbicides [10]. Duggleby and Pang reported that IMI binds to acetolactate synthase (ALS) and subsequently inhibits the synthesis of branched-chain amino acids (BCAA), particularly of valine (Val), leucine (Leu) and isoleucine (Ile) [11]. Imazethapyr (IM) is an ALS-inhibiting herbicide that has an asymmetric carbon atom, typically consisting of one pair of enantiomers, which can be separated by HPLC [12]. Zhou et al and Qian et al demonstrated that IM could enantioselectively inhibit rice or maize growth, and that *R*-IM showed a stronger inhibitory effect than *S*-IM [4], [13]. Furthermore, Zhou et al observed that *R*-IM inhibited the growth of maize by damaging the root morphostructure and ultrastructure more severely than that of *S*-IM [13]. By a molecular docking method, Zhou et al reported different modes of interaction by *R*- and *S*-IM with ALS [14].

Many publications have shown that biotic and abiotic stresses, such as low and high temperatures [15]–[17], UV irradiation [18], ozone [19], excess excitation energy [20], pathogen infection [19] and pesticides [21]–[23] lead to the overproduction of reactive oxygen species (ROS) in plants. ROS can destroy organelles, damage the membrane system and inhibit related gene expression. To cope with these ROS, plants have evolved intricate mechanisms to remove ROS by means of antioxidants and antioxidative enzymes. In fact, the reaction between antioxidants and ROS is known to occur in all living organisms [24], and life is a balance between them. Under optimal plant growth conditions, ROS are mainly produced at a low level in organelles (i.e., chloroplasts, mitochondria and peroxisomes), and antioxidants serve to reduce the levels of ROS, permitting them to perform useful biological functions without causing too much damage [25]. Under stress, electrons that have a high-energy state are transferred to molecular oxygen to produce ROS [26], [27], which damage proteins, DNA and lipids [28]. The main cellular enzymes/pathways that remove ROS from plants include: (1) superoxide dismutase (SOD) (e.g., in chloroplasts as part of the water-water cycle); (2) catalase (CAT) in peroxisomes; (3) glutathione peroxidase and its regenerating cycle (GPX); and (4) the ascorbate-glutathione (the Halliwell-Asada pathway, APX) pathway in the stroma, cytosol, mitochondria and apoplast. Although some reports have shown that *R*-IM damages the root morphostructure and ultrastructure, and that it inhibits gene expression more strongly than *S*-IM, little is known about the enantioselective response of IM on ROS formation and its effects on the antioxidant system.

Plant growth is significantly affected by carbohydrates produced via photosynthesis [29]. Besides used for biosynthesis of many important molecules such as proteins, nuclear acids and lipids, fixed carbon can also be temporally stored in the form of starch. Therefore, plants must achieve a balance between carbon assimilation and carbon storage and growth [30]. Although it is known that IM retards plant growth enantioselectively, we still do not know whether it is involved in disturbing carbon metabolism.

To gain a deeper insight into the enantioselectivity of IM in plants, and, in particular, to determine the interaction between oxidants and antioxidants, starch synthesis and degradation, we selected the model plant *Arabidopsis thaliana* for our study. Given that most antioxidant proteins are encoded by multigene families sharing high sequence identities, we used quantitative real-time PCR to sensitively and gene-specifically determine the mRNA levels of antioxidant enzymes, which included seven SOD genes, one CAT gene, six APX genes and eight GPX genes.

## Results

### Enantioselective effects of IM on plant growth

**Figure 1:**
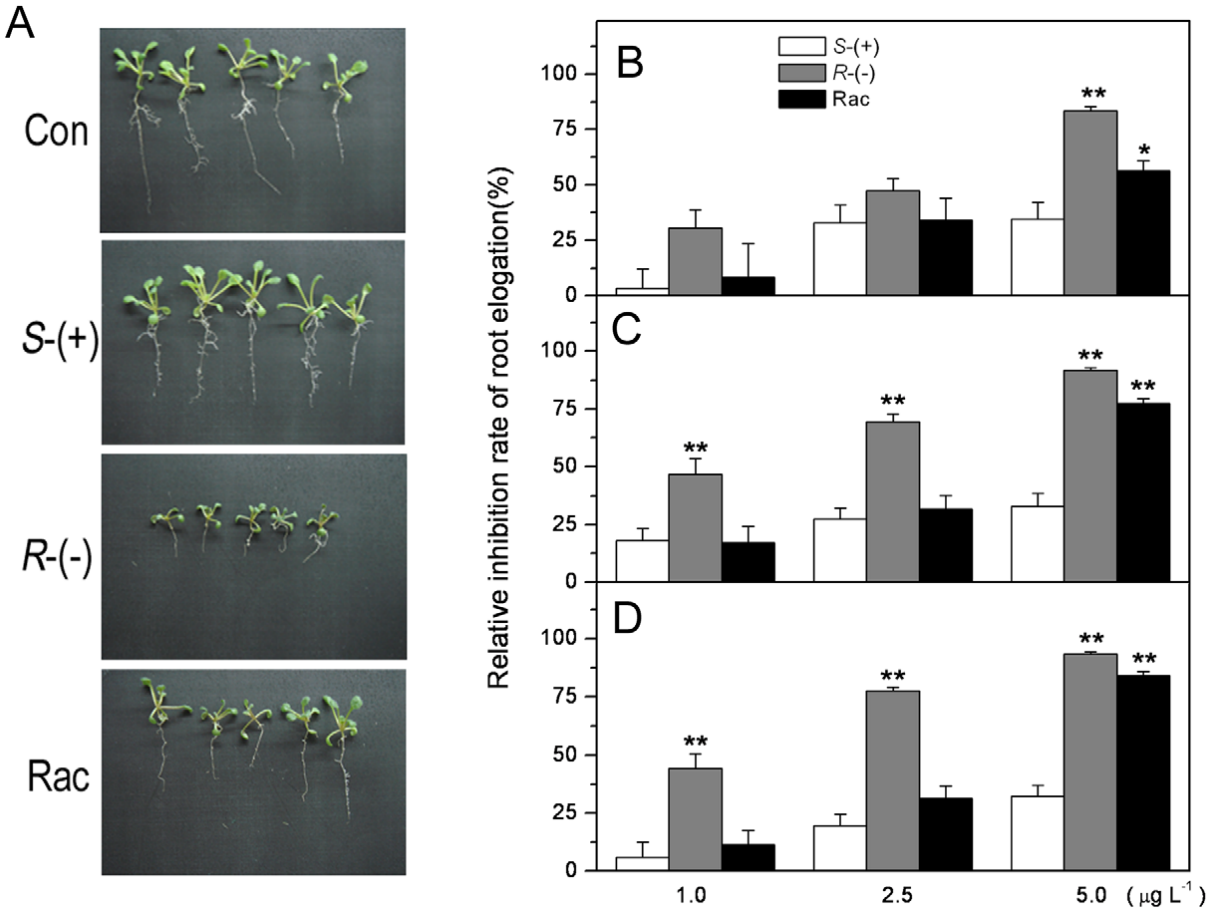
Phenotype of *Arabidopsis thaliana* plants subjected to IM-enantiomer treatment. **A**. Photograph of the control and plants treated by *R*-, *S*-IM and the racemic mixture; The relative inhibition rate of root elongation of *A. thaliana* is shown in **B–D**, after 2, 3 and 4 weeks of IM exposure, respectively. * represents a statistically significant difference (of *p*<0.05), when compared to that of *S*-IM exposed plants; ** represents a statistical significance at the *p*<0.01 level.

When plantlets were grown on the media containing *S*-IM, *R*-IM or racemate, a significant difference in plant growth was observed among treatments (Figure 1A). *R*-IM was more effective to inhibit plant growth than *S*-IM or racemate. Figure 1B, C and D showed root elongation of plants treated by the three tested concentrations of IM enantiomers and racemates after two, three and four weeks of exposure, respectively. We analyzed the relative inhibition rate of the root (RI) elongation and observed that RI increased in a dose-dependent manner of IM. At a concentration of 1 µg L^−1^, *R*-IM treatment showed the strongest inhibitory effect on roots, and the RI reached about 45% after 3 weeks of exposure. At the concentrations of 2.5 and 5.0 µg L^−1^ IM, the inhibitory effects of the enantiomers and racemates were stronger than that observed at 1 µg L^−1^. Among the enantiomers and racemates, *R*-IM showed the strongest inhibitory effect, and the maximum RI reached 93.4% while *S*-IM and racemate treatments reached to 34.4% and 83.9%, respectively. Based on this result, we selected 2.5 µg L^−1^ concentration of IM enantiomers in the following experiments.

The assessment of plant growth was also carried out by measuring the fresh weight (FW) of plantlets after exposure to the IM enantiomers (Table 1). The FW of plantlets in the control reached approximately 11.4 mg per plantlet after 3 weeks of growth, but slightly decreased in the *S*-IM treatment (16.7%) and drastically in the *R*-IM treatment (73.7%) and the racemic mixture (41.2%). A similar phenomenon was observed after 4-weeks exposure, but the decrease was more evident and only 32.8%, 75.2% and 50.4% of the control after *S*-IM, *R*-IM and racemate exposure, respectively.

**Table 1:** The effect of IM enantiomers on fresh weight (FW), malondialdehyde (MDA) and water content (WC).

	3 week			4 week		
	F.W. (mg/plantlet)	MDA (nmol/mg FW)	WC (%)	FW (mg/plantlet)	MDA (nmol/mg FW)	WC
Control	11.4±2.2	0.1±0.0	92.5±0.6	23.8±2.1	0.1±0.0	92.5±0.8
*S*-(+)-IM	9.4±1.2	0.1±0.0	92.5±0.7	16.0±1.3**	0.1±0.0	90.3±1.2
*R*-(−)-IM	3.0±0.5** ##	0.6±0.1** ##	87.4±0.6** ##	5.9±0.5** ##	0.6±0.1** ##	86.2±1.4** ##
Rac	6.8±0.6**	0.3±0.0*	91.9±0.2	11.8±0.8**	0.3±0.0*	91.6±1.2

* or ** indicates that the values are significantly different, as compared with the control (*p*<0.05 or 0.01, respectively).

## indicates that the values are significantly different, as compared with those of the *S*-IM-treated plants (*p*<0.01).

### The enantioselective effects of IM on ALS activity and amino acid synthesis

**Figure 2:**
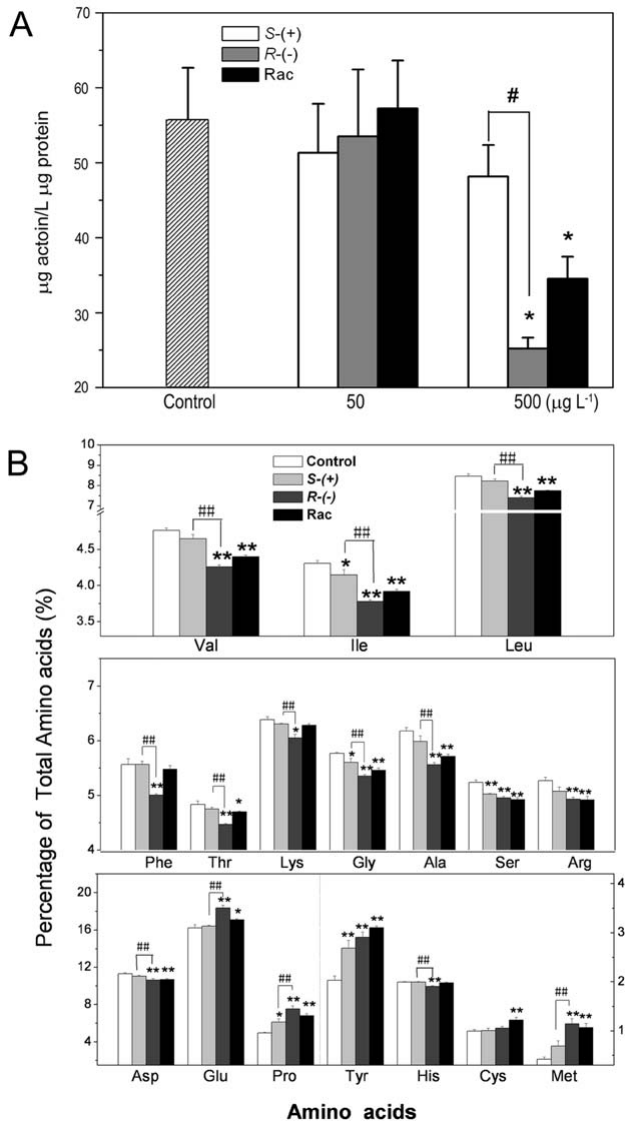
The effect of IM enantiomers on ALS activity *in vitro* and amino acid content. **A**. The effect of IM enantiomers on ALS activity in vitro; **B**. Amino acid content of *Arabidopsis thaliana* after 4 weeks of exposure. * or ** indicate that the numbers are significantly higher than those of the wild-type plants (*p*<0.05 or 0.01, respectively). # or ## indicate that the numbers are significantly different compared to those of *S*-IM-exposed plant (*p*<0.05 or 0.01, respectively).

Given that ALS is a target of IM, we measured the ALS activity in vitro after IM enantiomer exposure. As shown in Figure 2A, the concentration of 50 µg L^−1^ IM did not influence ALS activity in plants. When treated with a higher concentration of IM and its *R*- and *S*- enantiomers (500 µg L^−1^), the activity of ALS decreased to 61.9%, 45.2% and 86.5% of the control, respectively. This result is consistent with the above data showing that *R*-IM is the most effective in inhibition of plant growth.

Since ALS is the key enzyme in the synthesis of BCAAs, we measured content of amino acids. The content of Ile, Leu and Val decreased significantly after IM racemate and *R*-, *S*-IM treatment (Figure 2B); and only 87.7%, 87.5% and 89.5% of the control, respectively, in the *R*-IM treated group. The content of other amino acids (i.e., Phe, Thr, Lys, Gly, Ala, Ser, Arg, and Asp) in plants treated with IM also decreased compared with those in the control. Among them, *R*-IM also showed the strongest inhibition of biosynthesis of the above amino acids. In contrast, concentrations of Pro, Glu, Met and Tyr increased when plants treated by IM (Figure 2B). Proline accumulation was also reported in rice when treated by IM [4]. Accumulation of proline has been regarded as a marker for monitoring a plant response to environmental stresses as it acts as a stress-related signaling molecule [31], [32].

### The enantioselective effects of IM on superoxide radical and hydrogen peroxide accumulation

**Figure 3:**
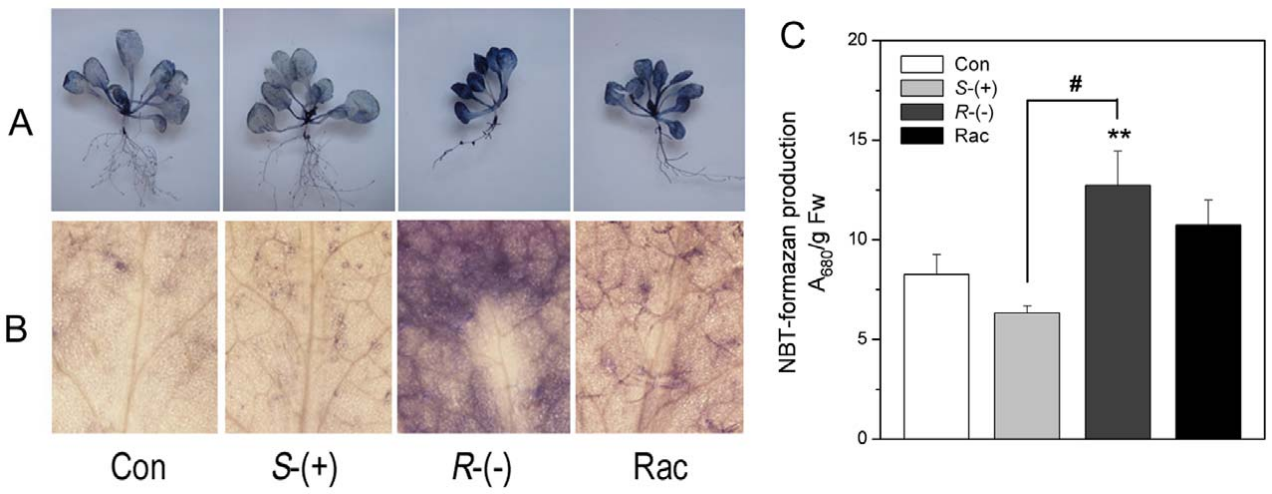
The superoxide anion accumulation after 4 weeks of IM-enantiomer treatment. **A**. A plantlet stained with NBT; **B**. A leaf stained with NBT; **C**. The colorimetric quantification of NBT-formazan production in plant extracts. ** indicates that the numbers are significantly higher than those of the control plants (*p*<0.01). # indicates that the numbers are significantly higher than those of *S*-IM-exposed plant (*p*<0.05). FW, fresh weight.

To investigate whether an oxidative stress is generated by treatment with IM enantiomers, superoxide radical (O_2_^−^) were examined by NBT-staining (nitroblue tetrazolium, NBT). As shown in Figure 3A and B, the deepest blue coloration was detected in plants treated with *R*-IM among all treatments. Quantification of the formazan spots demonstrated that *R*-IM-treated plants produced higher amounts of formazan precipitate than the control, *S*- IM or racemate (Figure 3C). These results suggest that *R*-IM has the strongest capacity to induce superoxide radicals.

**Figure 4:**
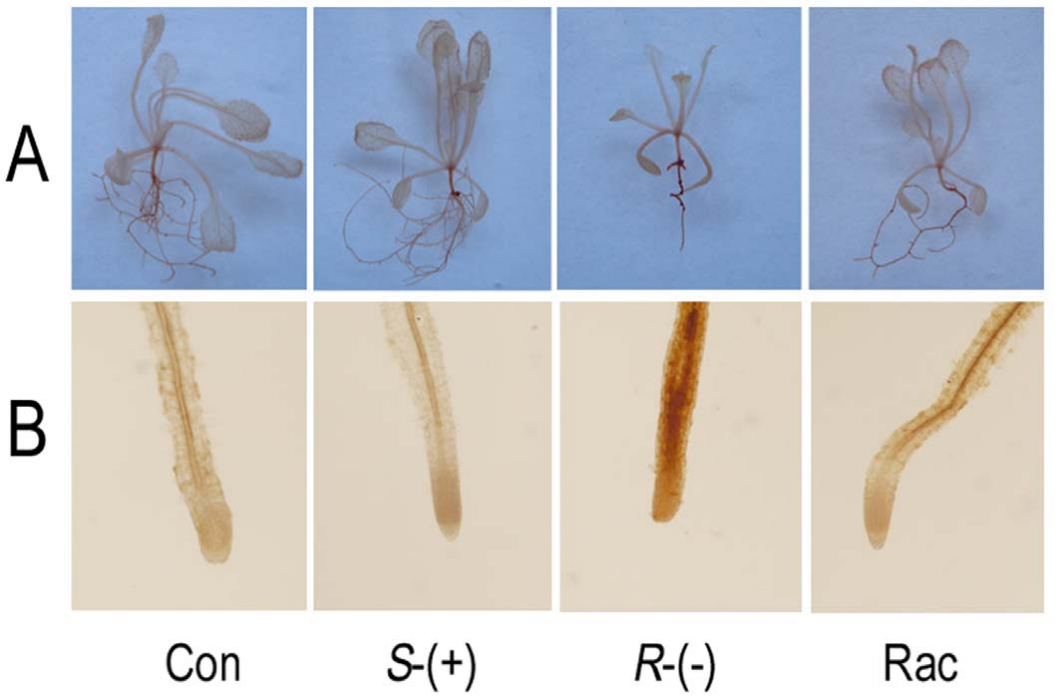
Hydrogen peroxide accumulation after 4 weeks of IM-enantiomer treatment. **A**. A plantlet stained with DAB; **B**. A root stained with DAB.

We also tested for hydrogen peroxide accumulation in IM enantiomer-treated plantlets using 3,3′-diaminobenzidine (DAB) staining, where an increase in the intensity of the red-brown stain is correlated with an increase in the H_2_O_2_ concentration. Unexpectedly, the intensity of red-brown staining in the leaf was not significantly affected by IM enantiomers (Figure 4). However, root tissue showed the strongest intensity of red-brown staining when plants treated by *R*-IM but not by *S*-IM. The red-brown color in roots treated with the IM racemate was of intermediate intensity (i.e., between the *R*-IM and *S*-IM levels). Because DAB staining relied on the presence of H_2_O_2_, we conclude that H_2_O_2_ was induced to the highest level by the treatment of *R*-IM, and H_2_O_2_ accumulation was more significant in the root than in the leaf.

### The enantioselective effects of IM on subcellular structure

**Figure 5:**
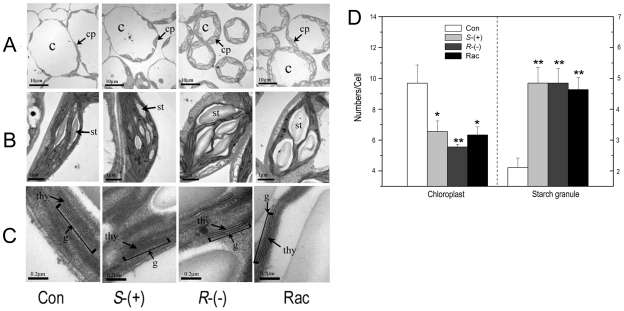
Changes of chloroplasts in *A. thaliana* after 4 weeks of IM-enantiomer treatment. **A**. Mesophyll cell structure; **B**. Chloroplast structure; **C**. Grana lamella structure; **D**. Number of chloroplast and starch granule *per cell*. cp, chloroplast; sg, starch granule; g, grana; thy, thylakoid. * or **represents a statistically significant difference of *p*<0.05 or 0.01, respectively, when compared to that of control.

The chloroplast is one of the main sources of ROS, and an increase of ROS may damage the organelle's structure. Therefore, the structure of the chloroplast subcellular membranes was investigated. Figure 5 A–C showed that IM had a marked effect on the number and structure of chloroplasts. The number of chloroplasts per mesophyll cell in one cut section decreased from 9.7 in the control cell to 6.6, 5.6 and 6.3 per cell after *S*-, *R*-IM and racemate treatment, respectively (Figure 5D). We also observed that the cell size was reduced (the vacuole also became smaller) after IM treatment, and chloroplasts appeared swollen and misshapen, which was especially evident after the *R*-IM treatment. To test whether this result was caused by water loss in the cell, we measured the water content (WC) in fresh plantlets. We observed that the value of WC in *R*-IM treatment was significantly lower than that of the control and *S*-IM treatment, while WC in the *S*-IM and racemate treatments did not change significantly (Table 1).

Figure 5B and D show that starch granules increased to approximately five granules per chloroplast in one cut section in the three treatment groups, as compared with two granules in the control. In many cells, the increased starch occupied almost the entire chloroplast after *R*-IM and racemate exposure. We also observed the structure of the grana lamella and found that this structure did not change noticeably after *S*-IM treatment, but it did become thin or partially disrupted after *R*-IM treatment (Figure 5C).

### The enantioselective effects of IM on the starch and sugar contents

**Figure 6:**
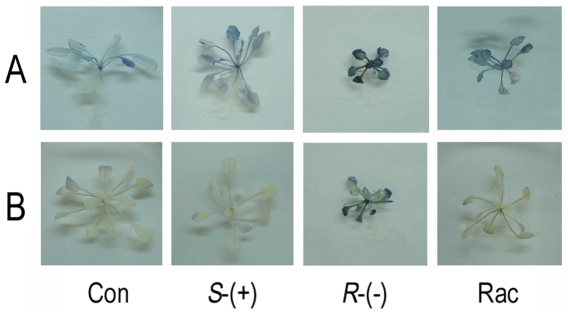
Plants stained for the presence of starch with iodine after 4 weeks of IM exposure. **A**. Plants harvested at the end of the light; **B**. Plants harvested at the end of the dark.

Given that starch granules increased after the treatment with *R*-IM, we stained for starch with an iodine solution at the end of the light and dark cycles. The leaves of plants exposed to *R*-IM were stained the darkest at the end of the light cycle, which indicated that they contained a significant amount of starch. The racemate-exposed plants stained slightly, as compared with the control, and the *S*-IM-treated plants stained with approximately the same intensity as the control. We also examined the leaves at the end of the dark period and found that the staining was very light, which indicated that most of the starch was degraded after the dark period in the *S*-IM and racemate treatments and in the control. Conversely, the leaves treated with *R*-IM stained very darkly, which indicated that a significant amount of starch was not degraded in these leaves (Figure 6).

**Figure 7:**
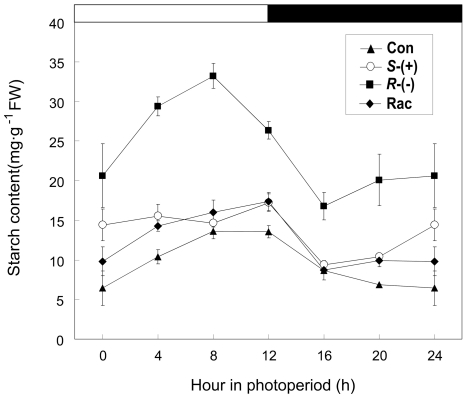
The diurnal changes of the starch content after 4 weeks of IM exposure. The white and black solid bars indicate the time of the light and dark period, respectively.

In order to further investigate the change in the starch content during the diurnal cycle, we measured the starch content at specific time points (Figure 7). The control plants displayed a steady rate of starch accumulation in the light, which reached a peak at the end of the light and then began to decline, reaching a minimum at the end of the dark period; these results agree with previous reports [33], [34]. *R*-IM-exposed plants also showed starch accumulation in the light, which reached a peak (approximately 33.22 mg g^−1^ FW) before the end of the light period. The starch decreased before the dark period, and the lowest starch content was approximately 16.77 mg g^−1^ FW. Compared with other treatments and the control, the *R*-IM-exposed plants had much higher levels of starch all day (about 2.5-fold compared with the control), even at the end of the dark cycle, indicating that a considerable amount of starch was not degraded, which was in complete accord with the staining result. The trend of the change in starch content in plants treated with *S*-IM and the racemic mixture was similar to the control; however, the starch content in those treatments (i.e., *S*-IM and racemate) were a bit higher than in the control, a result which was also observed in the TEM and iodine staining.

**Figure 8:**
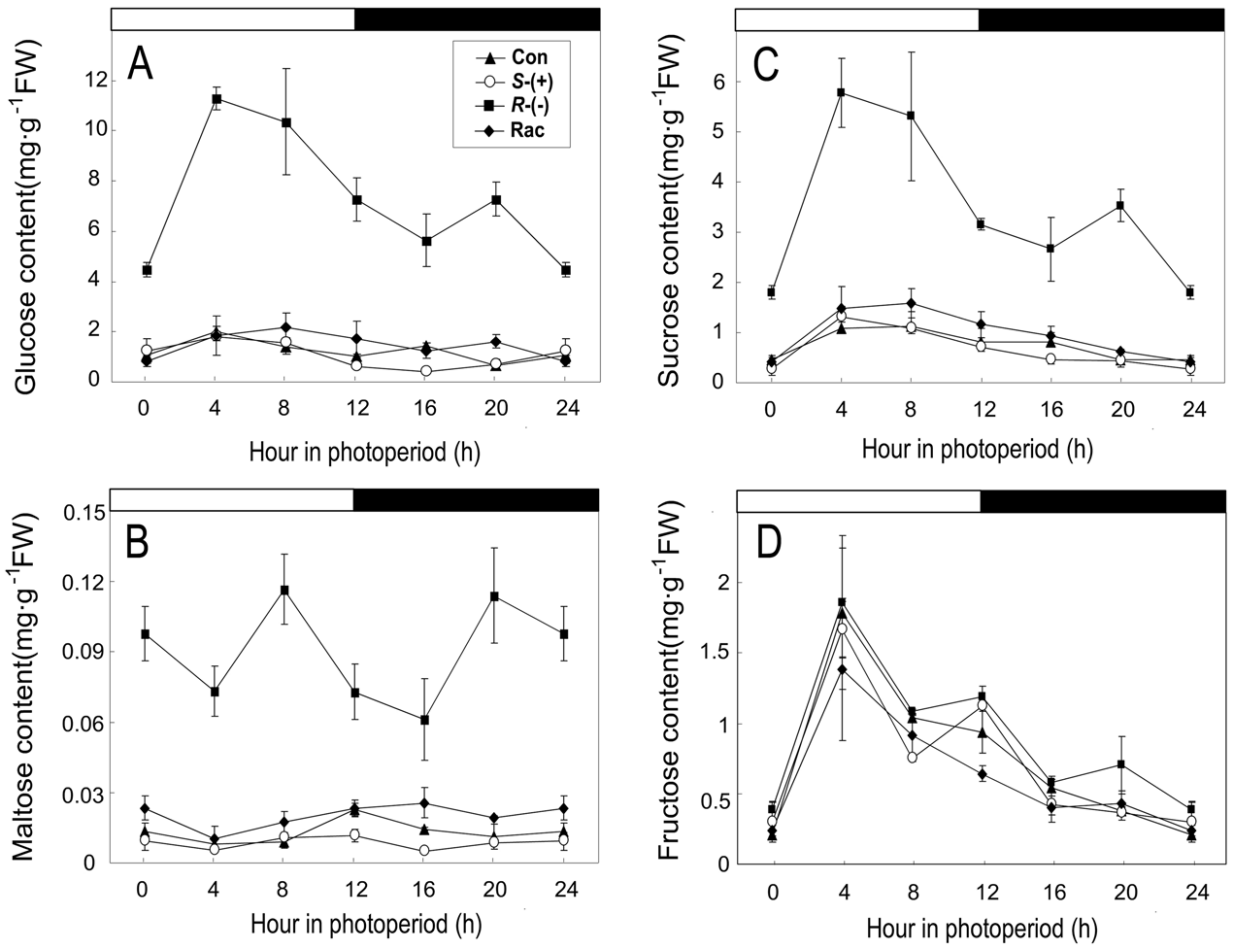
The diurnal changes of glucose, maltose, sucrose and fructose content after 4 weeks exposure. **A**. glucose content; **B**. maltose content; **C**. sucrose content; **D**. fructose content. The white and black solid bars indicate the time of the light and dark period, respectively.

Starch accumulates in the light as carbon storage and is degraded into sugar to maintain plant growth in the dark. Therefore, we measured the change of glucose, maltose, sucrose and fructose contents during the diurnal period. The glucose content maintained a relatively stable level both in the light and dark periods in the control, *S*-IM and racemate-treated groups. However, the glucose content in the *R*-IM group increased 3.3- to 10.8-fold, compared with that of the control, and it was accompanied by an evident wave upon exposure to light; it increased quickly after transfer to the light period and subsequently decreased after 4 h of illumination until the onset of the dark period (Figure 8A). The change in the maltose and sucrose contents after IM exposure was similar to that of glucose: *R*-IM exposure induced an increase of the maltose and sucrose contents to 3.2–13.3-fold and 2.5–5.4-fold of the control, respectively. The content of maltose in the *R*-IM group exhibited an obvious wave and increased to its peak after 8 h of illumination; the maltose content then decreased sharply and reached a minimum after 4 h of dark treatment. Subsequently, the maltose content increased to higher levels. The content of sucrose in the *R*-IM group also showed a significant wave, and it increased to its peak after 4 h of illumination; the sucrose content then decreased and reached a minimum at the end of the dark treatment (Figure 8B, C). The contents of maltose and sucrose in the treatment of the racemic mixture were a little higher than in the control but did not change after *S*-IM treatment. The content of fructose did not show a significant difference among the treatments (Figure 8D). These results suggest that carbon metabolism is also significantly altered along with nitrogen metabolism in IM-treated plants.

### The enantioselective effects of IM on antioxidant gene relative transcript levels

**Figure 9:**
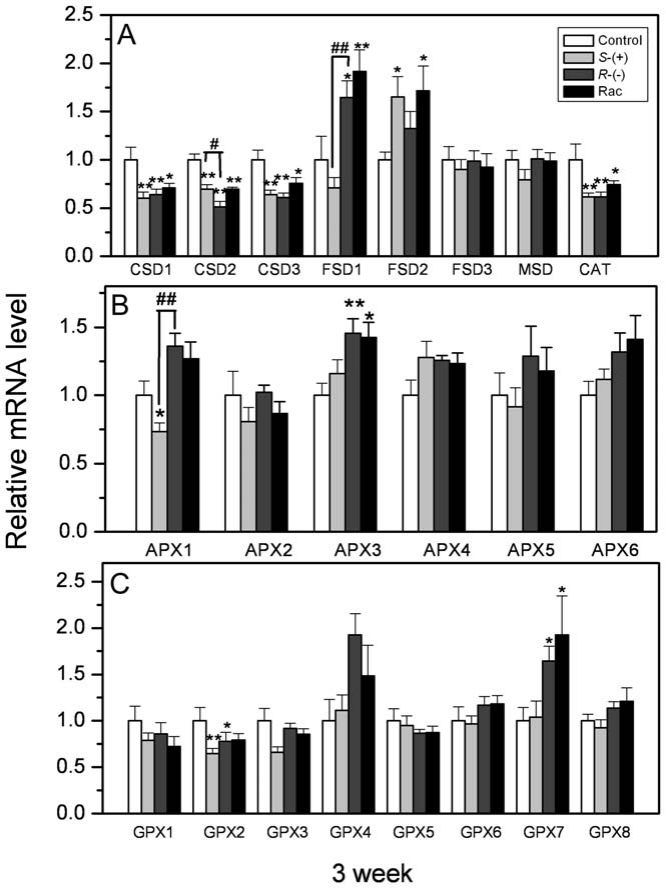
The gene expression of antioxidant enzyme in *A. thaliana* after 3 weeks of IM exposure. **A**. Gene expression of superoxide dismutase (SOD) and catalase (CAT); **B**. Gene expression of ascorbate peroxidase (APX); **C**. Gene expression of glutathione peroxidase (GPX). Values were normalized against actin 2 as housekeeping gene, and represent relative mean mRNA expression value ±SEM of 3 individuals. * or ** represents a statistically significant difference when compared to that of the control (*p*<0.05 or 0.01, respectively). # or ## represents a statistically significant difference when compared to *S*-IM-exposed plants (*p*<0.05 or 0.01, respectively).

One of main objectives of this work was to determine the effects of IM enantiomers on the expression of the antioxidant enzymes of *A. thaliana*. The first group of enzymes studied was the SODs, which are metalloenzymes that catalyze the conversion of O_2_^·−^ into O_2_ and H_2_O_2_. There are three types of SODs in *A. thaliana* that differ both in the metal cofactor at their active site and in their subcellular localization, as follows: cytosolic CuZnSOD (CSD1), thylakoidal CuZnSOD (CSD2), peroxisomal CuZnSOD (CSD3), thylakoidal FeSOD (FSD1, also located in mitochondria, the plasma membrane and chloroplast envelop), two chloroplast FeSODs (FSD2 and FSD3, also located in the chloroplast nucleoid), and mitochondrial MnSOD (MSD1). Based on the results of root growth (Figure 1), we selected a treatment of 2.5 µg.L^−1^ of IM to analyze the transcription of antioxidant genes. Figure 9A shows the effects of IM enantiomers on the relative transcripts of SOD genes after three weeks of exposure. The transcript levels of CSD1, CSD2 and CSD3 decreased significantly after IM exposure, and the decrease of CSD2 transcript was more evident after *R*-IM treatments and showed enantioselectivity. The transcript levels of FSD1 and FSD2 increased significantly, and FSD2 expression showed enantioselectivity after *R*-, *S*-IM and racemic mixture treatments. The transcript levels of other SOD genes did not change noticeably.

The expression of critical enzymes involved in the scavenging of H_2_O_2_ was also studied under IM-enantiomer treatment. CAT converts H_2_O_2_ to H_2_O and O_2_, and is localized in the cytosol, chloroplasts, mitochondria and peroxisomes. The transcript levels of the CAT gene decreased to 62%, 62% and 74% after *R*-, *S*-IM and racemic mixture treatments (Figure 9A), as compared to the control, but did not show enantioselectivity. Ascorbate peroxidases (APXs) are also key enzymes that scavenge hydrogen peroxide in plant cells. In this study, six types of APX were analyzed, which included three cytosolic (APX1, APX2, APX6) and three microsomal (APX3, APX4, APX5) enzymes. Compared with that of the control, the transcript levels of APX2, APX4, APX5 and APX6 were not affected significantly by IM treatment. However, the expression of APX1 decreased after *S*-IM exposure and showed enantioselectivity. In contrast, the abundance of APX3 transcript increased after *R*-IM and racemate treatment (Figure 9B). Glutathione peroxidases (GPXs) are a group of enzymes that catalyze the reduction of H_2_O_2_ in the presence of glutathione (the hydrogen donor). Milla et al (2003) identified seven GPX genes in *Arabidopsis thaliana*, and we used this molecular information to quantify their mRNA levels in IM-exposed plantlets [35]. However, we found that the transcript levels of GPXs did not change significantly, except that GPX2 decreased appreciably after IM exposure, and GPX7 increased after *R*-IM and racemate treatment (Figure 9C).

**Figure 10:**
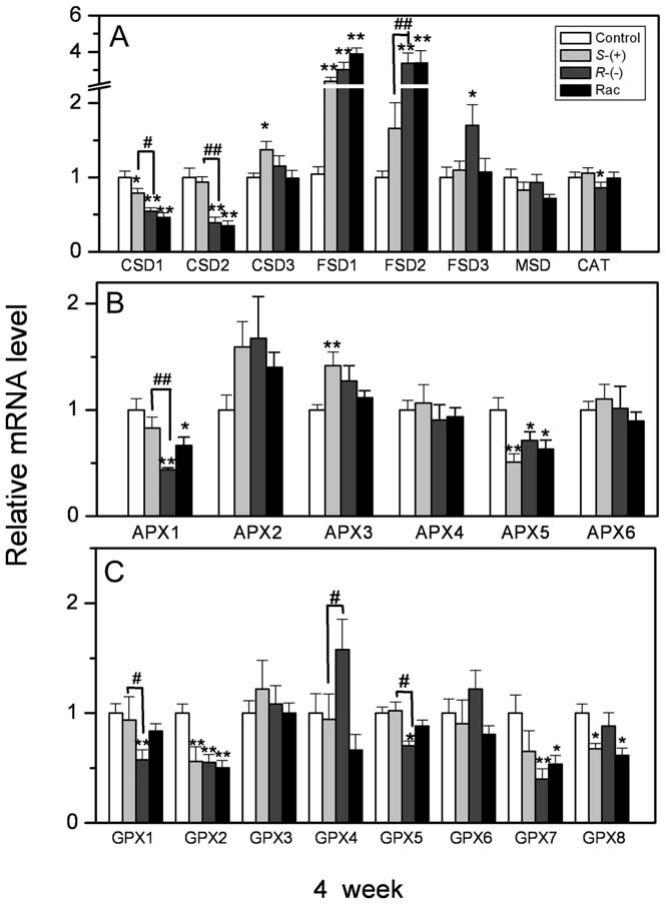
The gene expression of antioxidant enzyme in *A. thaliana* after 4 weeks of IM exposure. **A**. Gene expression of superoxide dismutase (SOD) and catalase (CAT); **B**. Gene expression of ascorbate peroxidase (APX); **C**. Gene expression of glutathione peroxidase(GPX). Values were normalized against actin 2 as housekeeping gene, and represent relative mean mRNA expression value ±SEM of 3 individuals. * or ** represents a statistically significant difference when compared to that of the control (*p*<0.05 or 0.01, respectively). # or ## represents a statistically significant difference when compared to *S*-IM-exposed plants (*p*<0.05 or 0.01, respectively).

Figure 10 shows the effects of the IM enantiomers on the relative transcript levels of antioxidant genes after 4 weeks of exposure. The transcript levels of CSD1 and CSD2 genes were down-regulated and showed enantioselectivity, while FSD1 and FSD2 increased significantly and reached 3.9- and 3.4-fold of the control, respectively. The transcript levels of FSD3 increased to 2.1-fold of the control after *R*-IM exposure, but it did not change after *S*-IM or racemate treatment. Similarly, the transcript level of CAT was also down-regulated significantly only by *R*-IM exposure (Figure 10A). The transcript level of APX1 was down-regulated after the treatment of *R*-IM and the racemic mixture to a degree that was 44% and 67% of the control, respectively, but it was not affected by *S*-IM. The transcript level of APX3 was up-regulated after *S*-IM exposure, APX5 was down-regulated by IM enantiomers exposure, and other APX genes were not affected (Figure 10B). The transcript levels of GPX1 and GPX5 were down-regulated after *R*- IM exposure and showed enantioselectivity. The transcript levels of GPX2, GPX7 and GPX8 were also down-regulated to some degree after IM-enantiomer treatment, whereas other GPX genes did not visibly change (Figure 10C).

### The enantioselective effects of IM on antioxidant enzyme activities

**Figure 11:**
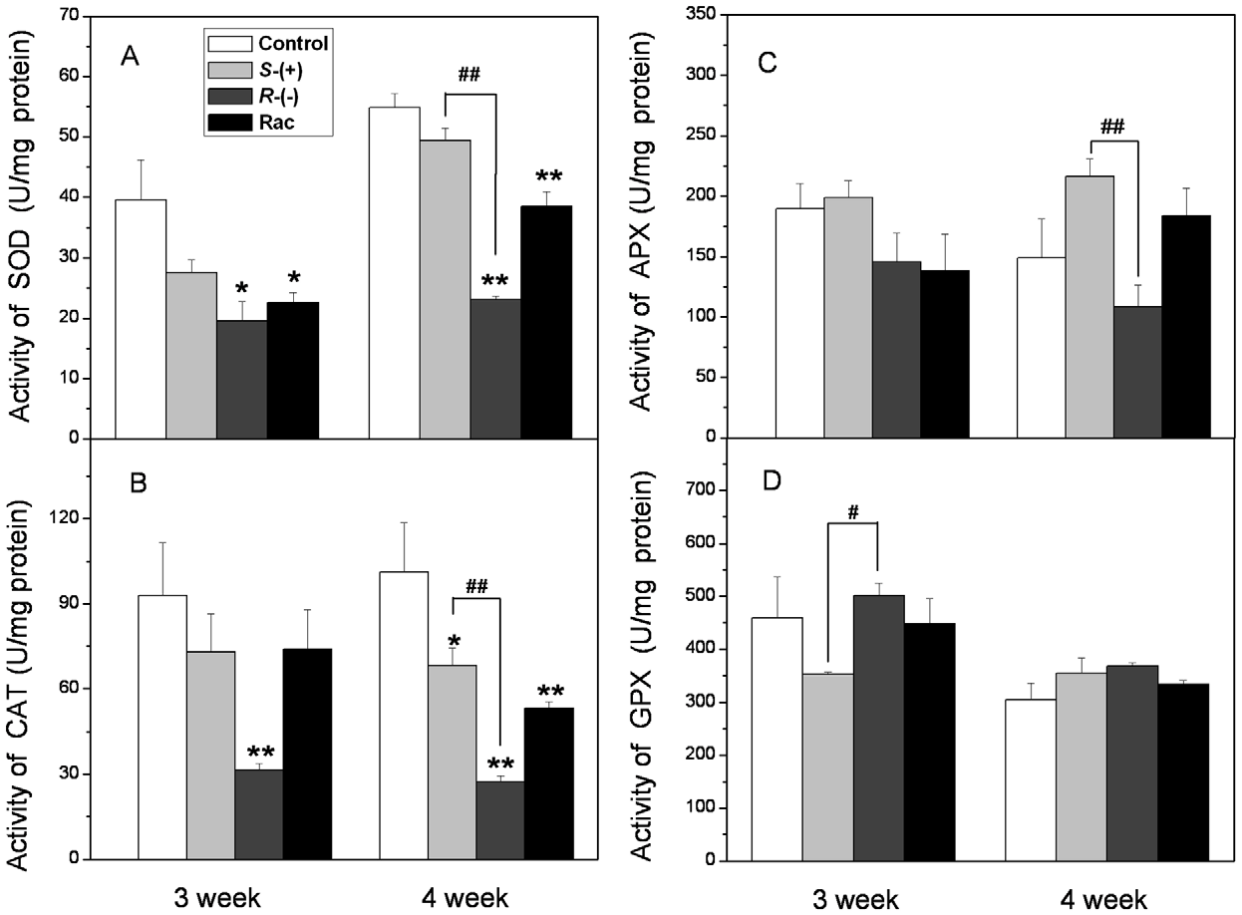
The activity of antioxidant enzyme in *A. thaliana* after 3 and 4 weeks exposure. * **A**. The activity of superoxide dismutase (SOD); **B**. The activity of catalase (CAT); **C**. The activity of ascorbate peroxidase (APX); **D**. The activity of glutathione peroxidase(GPX). * or ** represents a statistically significant difference when compared to that of the control (*p*<0.05 or 0.01, respectively). # or ## represents a statistically significant difference when compared to *S*-IM-exposed plants (*p*<0.05 or 0.01, respectively).

The effect of IM priming on the activity of antioxidant enzymes was studied in plantlets in order to compare it with the changes in antioxidant gene expression. We also evaluated the activities of antioxidant enzymes in 3- and 4-week-exposed plantlets. The activity of SOD is shown in Figure 11A; it decreased significantly after *R*-IM and racemate treatment but did not change perceptibly after *S*-IM exposure. The lowest activity of SOD was only 42.1% of the control, which was measured in plants after *R*-IM exposure. Measurements of CAT activity also indicated some significant changes following the treatment with IM; after 3 weeks of exposure, CAT activity decreased very significantly and was only 33.9% of the control after the *R*-IM exposure. *S*-IM and racemate treatment did not affect CAT activity significantly. However, CAT activity in the 3 treatment groups all decreased after 4 weeks of exposure, and the lowest CAT activity (*R*-IM treated group) was only 27.0% of the control (Figure 11B). Surprisingly, IM treatment did not affect APX or GPX activity (Figure 11C, D).

Finally, we measured the levels of malondialdehyde (MDA), which are indicative of lipid peroxidation as a marker of oxidative stress. The content of MDA increased by 4.6- and 1.8-fold after 3 weeks of *R*-IM and racemate exposure, respectively, but did not change after *S*-IM exposure. The change of MDA levels also showed the same pattern after four weeks of exposure (Table 1).

## Discussion

In this work, we investigated the effects of the enantioselective phytotoxicity of imazethapyr on the oxidant system and starch metabolism in *Arabidopsis thaliana* at the physiological and molecular levels. We used root length as an index of growth, because root length is an important agronomic trait and is easily affected by environmental stresses [36], [37]. The inhibition of root growth was more obvious as the treatment concentration increased, and also exhibited enantioselectivity. *R*-IM, *S*-IM and the racemic mixture all inhibited the Arabidopsis growth, as demonstrated in other plant species [4], [13]. Our results showed that *R*-IM has the strongest inhibitory effect on while *S*-IM has the lowest effect the growth of roots.

We also provided several lines of evidence supporting enantioselective effects of IM on the antioxidant system. We first evaluated the formation of ROS by a staining method and found that IM could stimulate ROS overproduction. It has been known that abiotic or biotic stress usually resulted in ROS overaccumulation [27]. ROS have been regarded as a signal that regulates plant growth, cell cycle, programmed cell death and cellular responses to biotic and abiotic stresses [36], [38]. We observed that *R*-IM induced the elevation of O_2_^.−^ and H_2_O_2_ levels more strongly than *S*-IM and the racemate (Figure 3). Then we examined the MDA content, an indicator of lipid peroxidation, and observed that *R*-IM treatment indeed resulted in MDA levels that were higher than *S*-IM (Table 1). Subsequently, TEM revealed that chloroplasts looked small, especially after *R*-IM treatment (Figure 5). The grana thylakoids became thin, and the integrity of a few membranes maybe disrupted; Mittler et al and Liu et al have reported that overproduction of ROS can initiate a variety of oxidative reactions on membrane unsaturated fatty acids, thus leading to the destruction of organelles and macromolecules [39], [40].

Based on previous reports, oxidative stress may be induced by an imbalance between ROS accumulation and scavenging factors, such as antioxidant enzymes. In this study, we aimed to analyze whether the expression of antioxidant genes changed following ROS production. Given that these antioxidant enzymes are encoded by multigene families, and that RNA gel blots are not amenable for the quantification of mRNAs [41], we utilized real-time PCR to analyze more than 20 genes encoding antioxidant enzymes in *Arabidopsis thaliana*. However, the transcription of these genes was complex and did not show a regulatory mechanism similar to that reported by Rubio et al [41]. Approximately ten of the antioxidant enzyme genes did not show either a significant change or enantioselectivity. Two of the genes showed an increase in transcript levels after IM exposure, and approximately ten antioxidant enzyme genes decreased after IM exposure and showed enantioselectivity. Transcription of these down-regulated genes was affected by *R*-IM exposure more strongly than by *S*-IM exposure. The activities of CAT, the SODs and APXs also decreased after IM exposure and were more affected by *R*-IM exposure than by *S*-IM exposure. These results demonstrate that ROS scavenging systems are rather suppressed, and ROS are enhanced, by IM exposure, as has been reported for others stress treatments [28], [42], especially *R*-IM.

It is easy to understand that herbicides may cause a reaction in the antioxidant pathway; several prior reports have proven that the target of IM is ALS *in vivo* and *in vitro* [43]–[45]. Our results demonstrated that the activity of ALS was inhibited enantioselectively by IM enantiomers in Arabidopsis. We also measured the amino acid content and found that levels of the three BACC decreased after IM exposure, especially following *R*-IM treatment; however, the decrease of BACC levels was less than 15%. If ALS is the sole target, can the decrease of BACC levels cause so evident a retardation of growth? Taking into account the results of TEM, where we observed the accumulation of many starch granules (to different degrees) in chloroplasts after IM-enantiomer exposure, we speculated whether carbohydrate biosynthesis or metabolism were also affected enantioselectively. Starch is one of the major products of photosynthesis in higher plants; it builds up in chloroplasts via the fixation of carbon during the day, and it is degraded to sugar during the night to sustain metabolism and growth [46], [47]. Gaston et al and Royuela et al have reported that imazethapyr caused starch or soluble sugar accumulation in pea, while Scarponi et al observed the contrary: IM significantly decreased starch content, but it increased glucose content, in soybean. In this study, we provided various evidence for the assertion that the starch content increased significantly and showed enantioselectivity after IM-enantiomer exposure [44], [48], [49]. Given that the accumulation and degradation of starch is controlled by circadian rhythms [50], we selected six specific time points, rather than one sampling point, to analyze the change in the starch and sugar content. In control plantlets, starch accumulated during the light period and reached a peak at the end of light exposure. Thereafter, the starch content began to decline, and by the end of dark period, was almost absent; however, the pattern of starch accumulation and degradation after *R*-IM exposure was impeded. Starch stopped accumulating before the end of the light cycle but was not degraded completely at the end of dark period. We analyzed the starch content from 8 d to 28 d by staining with iodine, and found that the starch accumulation at the end of dark period gradually increased from two-leaf plantlet (Figure S1). Starch degradation was clearly disturbed by *R*-IM; however, the pathway of starch degradation inside chloroplasts is relatively complex and requires the coordinated actions of a suite of enzymes [51]. We simply evaluated the expression of DSP4, which is the predominant phosphatase that is bound to chloroplast starch granules to control starch degradation (its down-regulation enhances starch accumulation), and we observed that the transcript levels of DSP4 significantly decreased after *R*-IM and racemate treatment (Figure S2).

Did the inhibition of starch degradation cause the decrease of sugar levels, which resulted in a shortage of a carbon source for growth and energy? To answer this question, we based our postulation of the following observation: that the carbohydrate content, including glucose, maltose and sucrose, in the *R*-IM treatment also accumulated to higher levels than in the control and the *S*-IM treatment. Therefore, we speculate that the degradation products (i.e., hexoses and phosphate) were not transported out of the chloroplast and further exported to heterotrophic tissues; thus, there was a lack of a carbon source for growth [30], [52], which resulted in an inhibition of plant growth after *R*-IM treatment. This phenomenon has been also found in the maltose transporter mutant (*mex1*) and glucanotransferase mutant (*dep2*), in which growth was clearly inhibited, and the plants accumulated high levels of starch and maltose [33], [47].

Sugar has been proved not only as a powerful driver of plant growth, but also as an effective signaling molecule [53]. High concentration of sugar inhibited chlorophyll accumulation and photosynthesis-related gene transcription [54]. Given C skeletons from sugar are necessary to synthesize amino acids, and plants process an intricate regulatory machinery to coordinate carbon (C) and nitrogen (N) assimilation, thus we analyzed whether transcript of key genes in C/N assimilation (i.e. Glutamate Receptor 1.1 (AtGLR 1.1), GS (Glutamine Synthetase) and GS2 (Glutamine Synthetase 2) [55], [56] was regulated by higher concentration of sugar (or regulated by IM enantioselectively). We observed that the transcript of these three genes did not change significantly by the treatment of either *R*- or *S*-IM (Figure S3), which meant sugar accumulation caused by IM did not regulate gene transcription in C/N assimilation enantioselectively. This result also suggested that the mechanisms by which sugars influence plant development and gene expression are complicated, and multiple sugar-response pathways and molecules actually being involved in the mechanisms might be not known in all cases.

In summary, as a chiral herbicide, IM affected Arabidopsis growth in an enantioselective manner. Doubtless, *R*-IM had a stronger herbicidal effect than *S*-IM. *R*-IM inhibited ALS activity that resulted in a decrease in the synthesis of various amino acids; it stimulated ROS formation, yet it decreased antioxidant gene expression and their enzymes activities, resulting in the disruption of membrane structure. Furthermore, another main cause of the enantioselective phytotoxicity that was observed for *R*-IM was a strongly disturbed carbohydrate utilization, which resulted in the almost complete cessation of plant growth.

## Materials and Methods

### Chemicals and Reagents

The racemic imazethapyr mixture (98% purity) was kindly provided by Shenyang Research Institute of Chemical Industry (Shenyang, China). Solvents used in the IM separation were HPLC-grade from Tedia (Fairfield, OH, USA). Enantiomers were separated according to Lin et al [12]. The imazethapyr enantiomers or racemic solutions were dissolved in acetone, with a final solvent concentration of 0.05% (v/v) for each experimental solution and the control.

### Plant materials, root length analysis and water content measurements

*A. thaliana* (ecotype Columbia [Col]) seeds were provided by Prof. Jirong Wang (National Laboratory of Plant Molecular Genetics, Institute of Plant Physiology and Ecology, Chinese Academy of Sciences). Seeds were sterilized with ethanol (75%) for 1 minute (min), extensively washed with distilled water and then sterilized with HgCl (0.1%) for 15 min. Sterilized seeds were vernalized at 4°C room for 2 days, and then germinated on agar plates with MS medium (supplemented with 30 g L^−1^ sucrose) and different concentrations of IM enantiomers or racemate in a culture room, equipped with cool-white fluorescence lights (approximately 300 µmol/m^2^/s) at a constant temperature of 25±0.5°C and a 12-hour (h) light/12-h dark cycle. Triplicate cultures were prepared for each treatment, every replicate contained at least five plantlets, and samples were taken after three and four weeks for RNA or enzyme extraction. The relative inhibition rate of root elongation caused by the IM enantiomers and racemate was determined the second week and calculated as previously reported [4]. The plantlets were dried at 95°C for 1 h to measure the water content (WC) by the following equation: WC (%) = 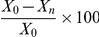, where 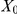 presents the average of the fresh weight and 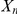 presents the dry weight. Three replicates were used for each treatment; every replicate contained five plantlets. According to the results of relative inhibition rate, 2.5 µg L^−1^ of IM enantiomers were selected in the following experiments.

### ALS activity measurements in vitro and the amino acid content analysis

Protein was extracted from four-leaf-stage plantlet tissue (5 g) without IM exposure. Tissue was frozen with liquid nitrogen and ground to a fine powder using pestle in the buffer containing 100 mM potassium phosphate buffer (pH 7.5), 1 mM sodium pyruvate, 5 mM MgCl_2_, 0.5 mM thiamine-pyrophosphate, 10 µM FAD and 10% (v/v) glycerol. Crude enzyme fraction was mixed with 50 and 500 µg L^−1^ (final concentrations) and reacted at 37°C for 90 min, and ALS activity measure was according to the method of Laplante et al [57].

Arabidopsis plantlets were collected after four weeks of IM exposure for amino acid measurement. Samples were; three replicates were used in each treatment. Approximately 300 mg of fresh plantlets were hydrolyzed in 5 ml of 6 N HCl under vacuum in an ampulla tube for 24 h at 110°C. The suspension was then filtered and evaporated under vacuum. The solid residue was dissolved in 2 ml of deionized water and evaporated twice again. The final residue was dissolved in 10 ml of 0.01 N HCl and filtered with a 0.45-µm filter membrane for the quantification of amino acids using an L-8800 automatic amino acid analyzer (Hitachi, Japan).

### Superoxide radical and hydrogen peroxide staining

The nitroblue tetrazolium (NBT) staining method of Rao and Davis (1999) was modified for the in situ detection of superoxide radicals [58]. After 4 weeks of IM exposure, the treated and control plantlets were immersed with a solution containing NaN_3_ (10 mM) and NBT (0.1% w/v) at 80°C for 20 min, the stained plantlets were bleached in 75% ethanol solution at 80°C for ∼5 min. Superoxide radical (O_2_^.−^) was visualized as a blue color produced by NBT precipitation. Superoxide radical quantification was according to the method of Myouga et al [59]. H_2_O_2_ accumulation in plants was visualized by 3, 3′-diaminobenzidine (DAB) staining, according to the method of Thordal-Christensen et al [60]. The plantlets were immersed in 1.25 mg ml^−1^ DAB, incubated on an orbital shaker for 18 h and then bleached in 95% (v/v) ethanol for 10 to 40 min to remove the chlorophyll. DAB is rapidly absorbed by plant tissue and is polymerized locally in the presence of H_2_O_2_ to yield a visible brown color.

### Subcellular structure detection by transmission electron microscopy

For microscopic analysis, samples of the control and 4-week IM-treated plantlets were fixed and embedded, according to previous reports [61]. For transmission electron microscopy (TEM), ultra-thin sections (70–90 nm) were prepared using a Reichert Ultracut ultramicrotome, stained with uranyl acetate followed by lead citrate and observed in a JEM-1230 microscope (Japan JEOL).

### Measurement of starch and sugar

For the measurement of starch and sugar, leaves were harvested at specified time points of the diurnal cycle from control and IM-exposed plants. Leaves were transferred to 80% ethanol and incubated in a boiling-water bath for 3 min (repeated three times) to remove pigments for starch measurement. Starch was digested with amyloglucosidase and α-amylase and then assayed for glucose [62]. Another part of leaves were boiled in water for 10 min and ground using pestle for sugar measurement. Supernatant was filter through 0.45 µm filter membrane and measured using high performance ion chromatography (ICS-3000, Dionex).

### RNA extraction, reverse transcription and real-time PCR analysis

*A. thaliana* tissues were collected and ground in liquid nitrogen to extract the RNA, according to the manufacturer's instructions (RNAiso™ Reagent, TaKaRa, Dalian, China). Reverse transcription (RT) was carried out using a reverse transcriptase kit (Toyobo, Tokyo, Japan); real-time PCR was performed with an Eppendorf MasterCycler® ep RealPlex^4^ (Wesseling-Berzdorf, Germany). Three Fe-SOD genes (FSD1, FSD2, and FSD3), three Cu/ZnSOD genes (CSD1, CSD2, and CSD3), one MnSOD gene (MSD1), one CAT gene (CAT), six ascorbate peroxidase genes (APX1 through 6) and eight glutathione peroxidase (GPX1 through 8) were selected and the primer pairs for each are listed in Table S1. The following PCR protocol was used with two steps: one denaturation step at 95°C for 1 min and 40 cycles of 95°C for 15 s, followed by 60°C for 1 min. Actin 2 was used as a housekeeping gene to normalize the expression profiles.

### Enzyme extraction and analysis

To extract antioxidant enzymes, *A. thaliana* plantlets were ground with 1.5 ml of 20 mM phosphate buffer (pH 7.4) in an ice bath. The homogenate was centrifuged at 10,000 g for 10 min at 4°C to obtain the supernatant used for assaying enzyme activities and MDA levels. The activity of SOD, CAT, and the level of MDA were determined as described previously [63]. APX enzyme was extracted and measured according to the method of Sun et al [64]. One unit of APX was defined as the amount of enzyme oxidizing one nmol of ascorbate per min. GPX activity was measured according to the GPX kit (Jiancheng Biotech., NanJing, China).

### Data analysis

Data are presented as mean ± standard error of the mean (SEM) and tested for statistical significance using the analysis of variance, which was performed with the StatView 5.0 program. Values were considered significantly different when the probability (*p*) was less than 0.05 or 0.01.

## Supporting Information

Figure S1 **Plants harvested at the end of the light and dark periods were stained for the presence of starch with iodine after 8 to 28 days of IM exposure.**
(TIF)

Figure S2 **The effect of IM enantiomers on the gene expression of DSP4 in** ***A. thaliana*** **after 4 weeks of exposure.** ** represents a statistically significant difference when compared to that of the control (*p*<0.01). # represents a statistically significant difference when compared to S-IM-exposed plants (*p*<0.05).
(TIF)

Figure S3 **The effect of IM enantiomers on the gene expression of AtGLR 1.1, GS and GS2 in** ***A. thaliana*** **after 4 weeks of exposure.** Different letter represents a statistically significant difference between them (*p*<0.05).
(TIF)

Table S1 **The sequences of primer pairs used in real-time PCR.**
(PDF)
