# Supplementary figures and images for: Enantioselective Phytotoxicity of the Herbicide Imazethapyr on the Response of the Antioxidant System and Starch Metabolism in Arabidopsis thaliana
Source: PLoS One. 2011 May 6;6(5):e19451. doi: 10.1371/journal.pone.0019451 (PMC3089624; doi:10.1371/journal.pone.0019451)

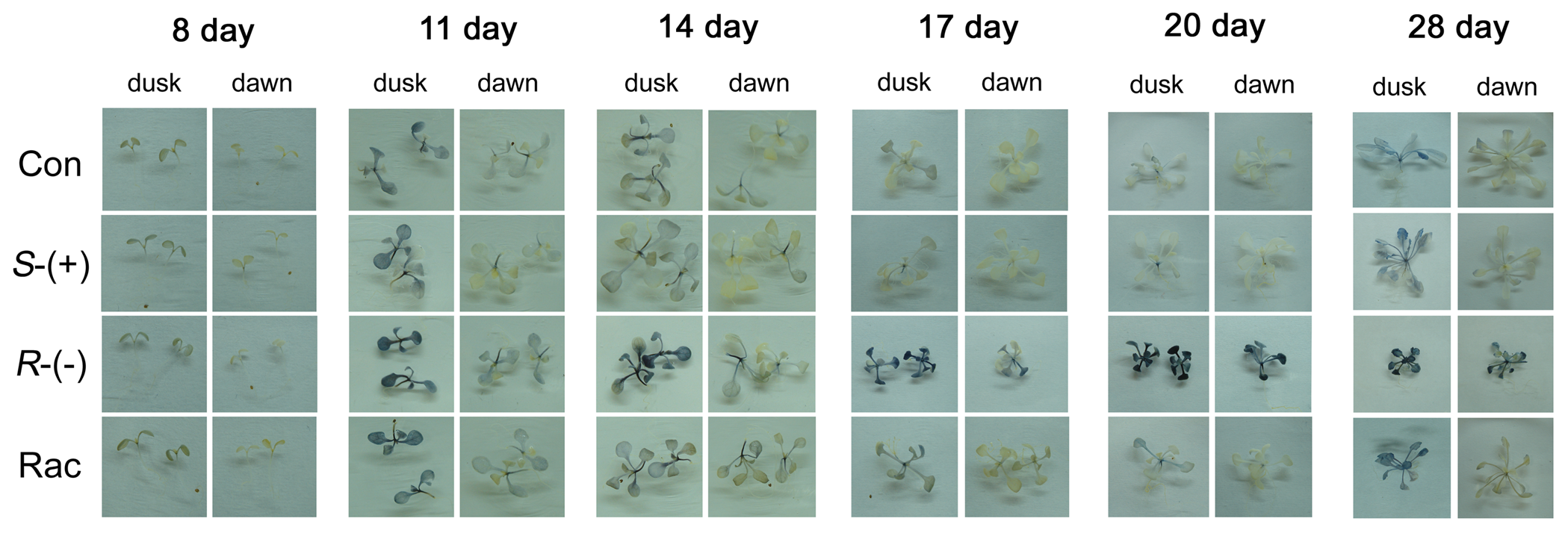

Supplement: Figure S1 — Plants harvested at the end of the light and dark periods were stained for the presence of starch with iodine after 8 to 28 days of IM exposure. (TIF) [file pone.0019451.s001.tif]

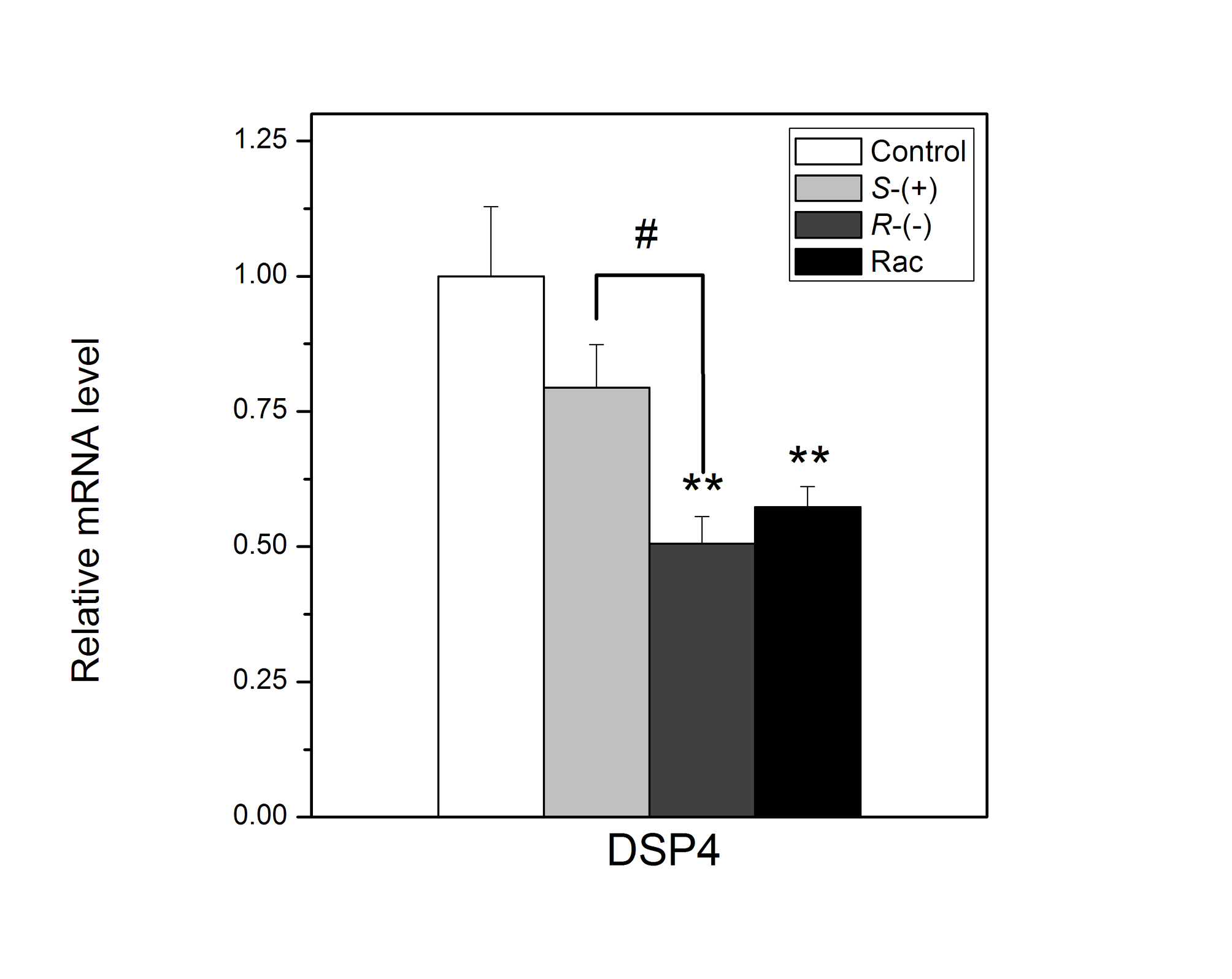

Supplement: Figure S2 — The effect of IM enantiomers on the gene expression of DSP4 in A. thaliana after 4 weeks of exposure. ** represents a statistically significant difference when compared to that of the control (p<0.01). # represents a statistically significant difference when compared to S-IM-exposed plants (p<0.05). (TIF) [file pone.0019451.s002.tif]

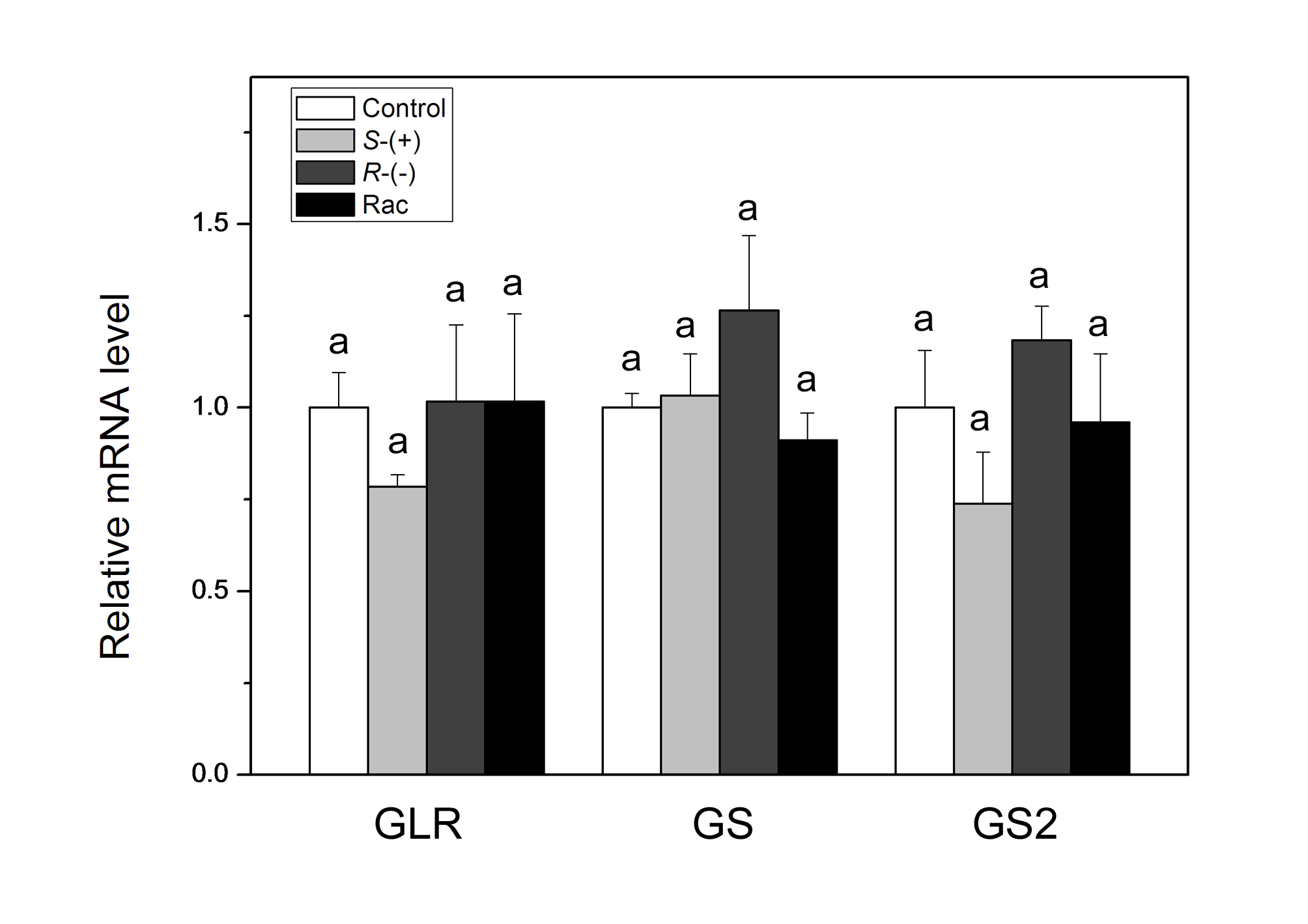

Supplement: Figure S3 — The effect of IM enantiomers on the gene expression of AtGLR 1.1, GS and GS2 in A. thaliana after 4 weeks of exposure. Different letter represents a statistically significant difference between them (p<0.05). (TIF) [file pone.0019451.s003.tif]
