# Supplementary material for: Enantioselective Phytotoxicity of the Herbicide Imazethapyr on the Response of the Antioxidant System and Starch Metabolism in Arabidopsis thaliana
Source: PLoS One. 2011 May 6;6(5):e19451. doi: 10.1371/journal.pone.0019451 (PMC3089624; doi:10.1371/journal.pone.0019451)
Supplement: Table S1 — The sequences of primer pairs used in real-time PCR. (PDF) [file pone.0019451.s004.pdf]

Enantioselective Phytotoxicity of the Herbicide Imazethapyr on the Response of the Antioxidant System and Starch Metabolism in *Arabidopsis thaliana*

Haifeng Qian, Tao Lu, Xiaofeng Peng, Xiao Han, Zhengwei Fu, and Weiping Liu

SUPPLEMENTARY DATA

Table S1. Primer sequences used in the real time qPCR analysis.

Gene name	Primer sequence	*Genbank accession no.*
Actin 2	F:ACCTTGCTGGACGTGACCTTACTGAT-3	Genbank :
	R:GTTGTCTCGTGGATTCCAGCAGCTT-3	NO.U41998.1
CSD1	F:TCCATGCAGACCCTGATGAC<br>R:CCTGGAGACCAATGATGCC	NM_100757.3
CSD2	F:CATTCTCATCTCCTTCTCG<br>R:GATTGGAGACGGTGGAGAT	NM_128379.3
CSD3	F:ACTGATGGAAGCTCCTAGA<br>R:GCCAGGAGAGAGTCCTGAGAT	NM_001125773.1
FSD1	F:CTCCCAATGCTGTGAATCCC<br>R:TGGTCTTCGGTTCTGGAAGTC	NM_179110.2
FSD2	F:GGAACCGCATATGAGCCGGGAAACC<br>R:CTTGTCTTCCTCCTTTGGGAGAGG	NM_124489.2
FSD3	F:GGTGGTTTAAAGGTTGAAGCTTACTACGG<br>R:CCTTATCAATCTGCTCAAGAACACCC	NM_122237.3
MSD1	F:ATGTTTGGGAGCACGCCTAC<br>R:AACCTCGCTTGCATATTTCCA	NM_111929.3
CAT1	F:AAGTGCTTCATCGGGAAGGA<br>R:CTTCAACAAAACGCTTCACGA	NM_101914.3
APX1	F:TGCCACAAGGATAGGTCTGG	NM_001123772.1

	R:CCTTCCTTCTCTCCGCTCAA	
APX2	F:TGATGTGAAGACGAAGACAGGAGGAC<br>R:CCCATCCGACCAAACACATCTCTTA	NM_111798.3
APX3	F:CCCAAAATCACATACGCAGACCTGTA<br>R:AGTTGTCAAACTTCAGCGGCTCTTG	NM_119666.3
APX4	F:CTACTAAATCCGGGGGAGCCAATG<br>R:CTCTGTTGCATCACTCCTTCCAAAAT	NM_116970.3
APX5	F:AGCTAAACCGTCCACACAACAAAGGT<br>R:GTCCCAAAGTGTGACCTCCAGAGAGA	NM_119763.2
APX6	F:TGCAAAACGAAATAAGGAAAGTGGTG<br>R:CACTCAGGGTTTCTGGAGGTAGCTTG	NM_119384.4
GPX1	F: CAGGAGGATTCTTGGGTGGTC<br>R: TTCTCGATTTGGAAAGGGGAT	NM_128065
GPX2	F: AAATGTGGTCTGACGGATGC<br>R: CATTGTTTCCTGGTTCTTGTCC	NM_128714
GPX3	F: AGTCAAGAGCCAGGCAGCAA<br>R: GCATCCCCAAACAATCCTCC	NM_129896
GPX4	F: GGGAAAGTCCTCCTCATCGTC<br>R: GTGCCGGGCTCCTGGTATA	NM_130382
GPX5	F: GGAAAGTGCTGCTCGTCGTC<br>R: GCAAGGAAACGCCAATACCA	NM_116173
GPX6	F: GCTCTTTGGAGACGGCATTA<br>R: AGGTGAGGTAGTTGGTGCGA	NM_117229
GPX7	F: ACACCCTTCAATGGTTTCGC<br>R: CTGAAACCCCATTACTCGGAT	NM_119337
GPX8	F: CAGTGAGAAATGGCGACGAA<br>R: CCCACATTTGGAAGCAACATT	NM_105025
